# Supplementary material for: Splice-Junction-Based Mapping of Alternative Isoforms in the Human Proteome
Source: Cell Rep. Author manuscript; Available in PMC 2020 Jan 15. (PMC6961840; doi:10.1016/j.celrep.2019.11.026)
Supplement: 2 [file NIHMS1546469-supplement-2.zip › DF1/PXD010154/Lung/Lung_14_EMSY_EGVSCSDEDEKPR.pdf]

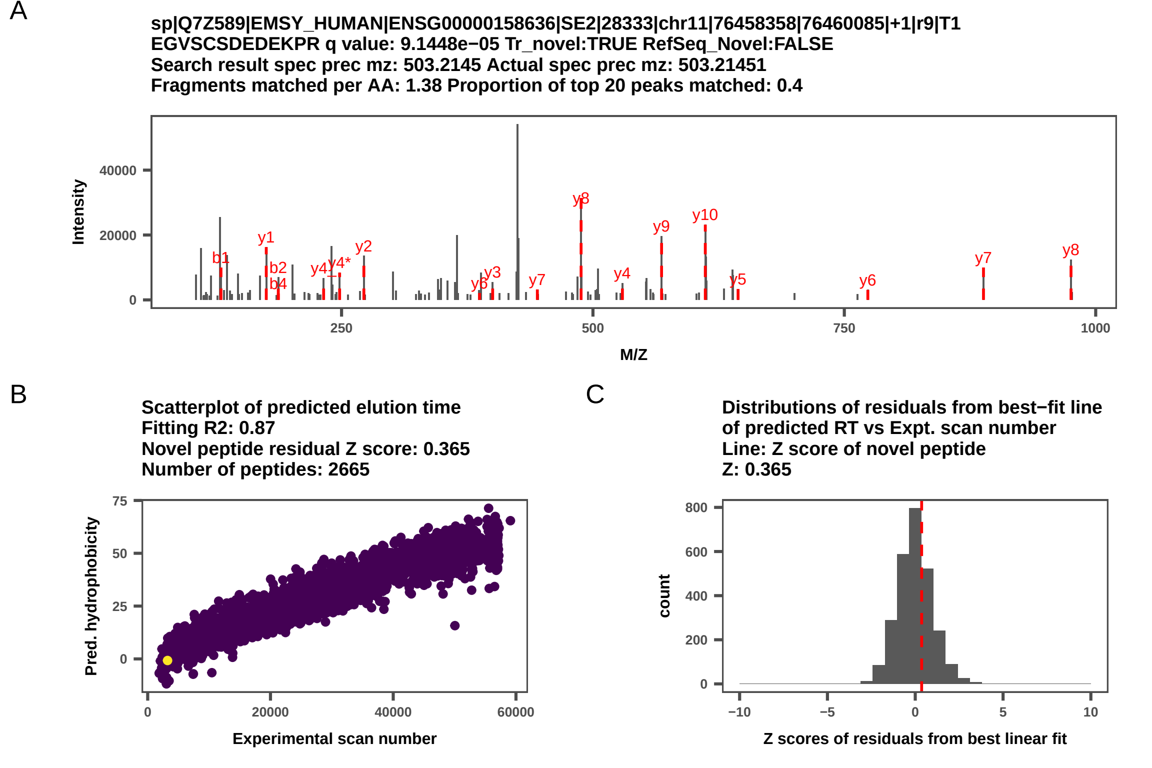
A
sp|Q7Z589|EMSY_HUMAN|ENSG00000158636|SE2|28333|chr11|76458358|76460085|+1|r9|T1
EGVSCSDEDEKPR q value: 9.1448e−05 Tr_novel:TRUE RefSeq_Novel:FALSE
Search result spec prec mz: 503.2145 Actual spec prec mz: 503.21451
Fragments matched per AA: 1.38 Proportion of top 20 peaks matched: 0.4
Intensity
40000
20000
0
b1
y1
b2
b4
y4_
y4*
y2
y6
y3
y7
y8
y4
y9
y10
y5
y6
y7
y8
250
500
750
1000
M/Z
B
Scatterplot of predicted elution time
Fitting R2: 0.87
Novel peptide residual Z score: 0.365
Number of peptides: 2665
Pred. hydrophobicity
75
50
25
0
0
20000
40000
60000
Experimental scan number
C
Distributions of residuals from best−fit line
of predicted RT vs Expt. scan number
Line: Z score of novel peptide
Z: 0.365
count
800
600
400
200
0
−10
−5
0
5
10
Z scores of residuals from best linear fit
